# Bonding Acetylated Veneer for Engineered Wood Products—A Review

**DOI:** 10.3390/ma15103665

**Published:** 2022-05-20

**Authors:** Maik Slabohm, Carsten Mai, Holger Militz

**Affiliations:** Wood Biology and Wood Products, Burckhardt Institute, Georg-August University of Göttingen, Büsgenweg 4, 37077 Göttingen, Germany; cmai@gwdg.de (C.M.); holger.militz@uni-goettingen.de (H.M.)

**Keywords:** acetylation, laminated veneer lumber, plywood, rotary cut veneer, wood modification

## Abstract

The purpose of this review is to put previous research findings on acetylated wood and the fabrication of veneer-based products in a common context. The first research on wood acetylation was already conducted in the 1920s using wood meal, whereas relevant research on veneer acetylation was published nearly two decades later, during the 1940s. In the years that followed, a great deal of research has been done on both solid wood and composite acetylation. Developments in the 1990s and early 2000s resulted in the creation of commercial products. Nowadays, wood is becoming increasingly popular in construction. Therefore, high-performance materials with high dimensional stability and durability are required. Veneers are thereby of particular relevance because of their propensity to absorb chemicals into even tough-to-treat wood species. However, acetylation alters the bonding properties of wood, which is important for the manufacture of engineered veneer products, especially in load-bearing construction. A large amount of research is now being conducted on the acetylation of veneer, and acetylated veneer products are anticipated in the near future. This study covers the fundamentals of bonding but focuses specifically on veneer acetylation and its fabrication to engineered veneer-based products. The influencing factors of acetylation on bonding are also discussed.

## 1. Introduction

Over 30% of the earth’s land area is covered with forests [1], with thousands of wood species. This source of biomass generates wood which is seen as one of the most sustainable “green” building materials on earth [2]. Wood has received a lot of attention in recent years as a natural and renewable resource that helps to mitigate climate change by long-term storage of CO_2_. Wood and its composites have a wide range of exterior applications, such as claddings and decking in everyday life, as exposed material in harsh maritime conditions, or as load-bearing construction material including multi-storey houses. For instance, in Norway, the “Wood Hotel” was recently raised to a height of 85.4 m as the tallest timber building in the world [3]. However, many European wood species have a low natural durability class [4] and cannot be utilized exteriorly without additional protection. Therefore, low durable wood species are usually enhanced by applying wood preservatives. Despite the fact that such wood preservatives improve resistance to biological decay, it is well recognized that some of them are detrimental to human health and the environment due to their biocidal activity [5,6,7].

Wood modification is an alternative approach for enhancing wood properties. It is a non-biocidal alteration of wood at the cell wall level by applying chemical, biological agents, or physical methods on the material to alter the wood characteristics, with the goal to have no or low release of toxic chemicals after modification, during service life, end of service life, recycling and disposal [8]. Various wood modification processes have been developed in recent decades, for instance, processes based on acetylation, furfurylation, treatment with curing resins (phenol-formaldehyde (PF), melamine formaldehyde (MF), 1.3-dimethylol-4.5-dihydroxyethyleneurea (DMDHEU), thermal treatments, among others, and some even to an industrial scale [8,9,10,11,12,13,14,15]. In that regard, acetylation is one of the few commercial chemical wood modification technologies available today, known as ACCOYA^®^ (Accsys Technologies S.A., Arnhem, The Netherlands)-based on solid wood and TRICOYA^®^ (Accsys Technologies S.A., Arnhem, The Netherlands)-based on wood fibers, respectively.

During acetylation, hydrophilic hydroxyl groups are esterified with acetic anhydride and acetic acid is formed as a by-product [8]. There is evidence that acetylation enhances the dimensional stability (moisture-induced shrinking and swelling) and durability (resistance to fungi and insects) of wood [8,11,12,14,15,16,17,18,19]. However, many European wood species cannot be adequately treated in larger dimensions, especially the heartwood parts which are more limited [4].

For this reason, another promising approach to solid wood and fiber acetylation is to treat thinner-dimension veneers. Thin veneers have the advantage of allowing chemicals to penetrate easily and uniformly into even difficult-to-treat wood species.

A single veneer has mostly no relevant application, except for research purposes. Therefore, it is either bonded on any kind of core board (for example chipboard) for decorative purposes or bonded together with other veneer sheets to plywood, LVL, and similar products. Thus, bonding plays a key role after acetylation of veneer, especially for load-bearing purposes. However, the bonding of wood is affected by acetylation.

This research puts veneer acetylation, engineered veneer production, and earlier discoveries on bonding acetylated solid wood into a common context. As a result, parameters that influence the bonding of acetylated veneer products were identified. Additionally, provided is a current market overview of acetylated veneer products. Various research needs evolved as an additional result of this investigation.

## 2. Bonding Wood

**Figure 1:**
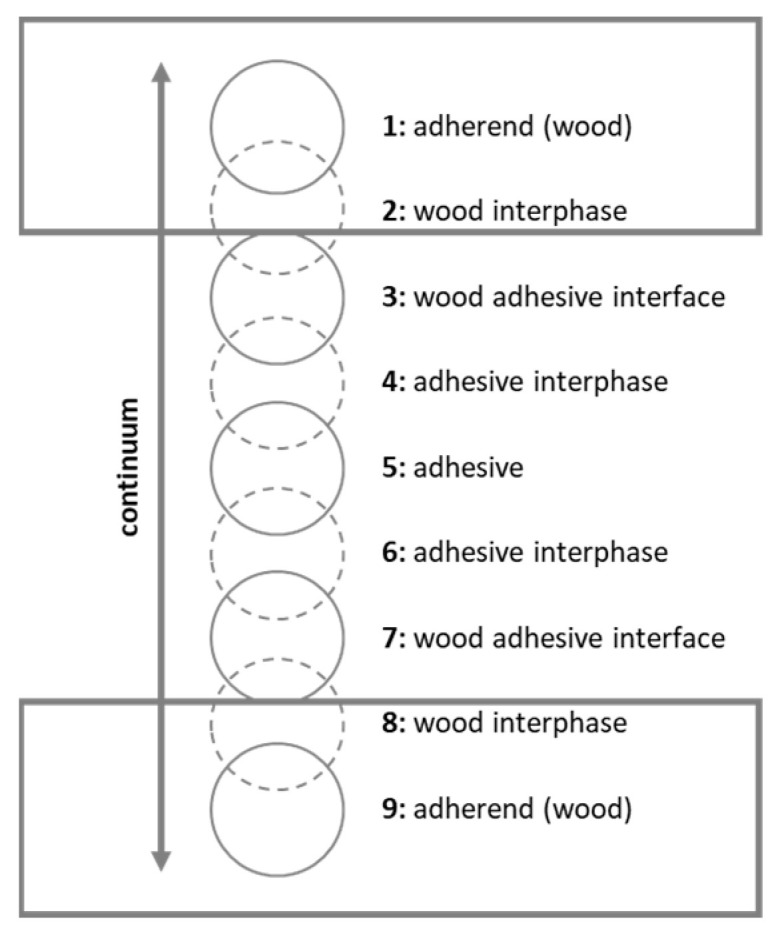
Own representation based on [21,22]. Marra [21] described the bonding as a nine-zone model. Frihart [22] stated that a continuum exists from adherend (1) through adhesive (5) to adherend (9), rather than discrete zones.

There is no basic theory of bonding, due to a large number of interacting factors. Cohesion is known as the interchange of atoms or molecules inside the same material [20] (for instance inside glue). Adhesion is the interchange of atoms or molecules of different materials inside the interphase (for example between glue and wood) (Figure 1). There are basically two types of adhesion: specific and mechanical. Specific adhesion describes chemical, physical, and thermodynamic interactions [20]. According to the same author, mechanical adhesion is defined as the penetration of the liquid adhesive into pores and other surface structures where the glue interlocks during curing.

The adherend wood (Figure 1) is depicted in a simplified form as a homogeneous substance. However, in practice, it is a heterogenous, orthotropic material and influences the bonding with its chemical (buffering capacity, pH, …), elasto-mechanical (elasticity, strength, …) and physical properties (density, grain angle, moisture, …) on macro-, micro- and sub-microscopic levels [23]. Thereby the adhesive and its proper application have a significant impact on the bonding [22]. The test method and subsequent use of the wood product are also crucial. There are numerous other factors that affect wood bonding, which are discussed in the literature [20,21,22,24,25,26].

## 3. Fabrication of Engineered Veneer Products

### 3.1. Veneer Processing

A veneer, by definition, is a thin sheet of wood. While rare and expensive veneers are often used for decorative purposes (for example furniture), other veneers are more commonly used in structural materials (plywood and LVL), special thin veneer strips, called micro-veneers, are often used in research [27,28,29,30,31,32,33].

Nevertheless, at an industrial scale, three different manufacturing processes are typically used: (1) rotary-cut or peeled veneer (for instance centric or eccentric), (2) sliced (for example quarter-sliced veneer), and (3) sawn [34,35]. The commercial production of plywood and LVL only requires rotary-cut veneers. During the peeling process, checks (or peeling checks, lathe checks) form due to the separation of wood cells that normally do not extend through the veneer thickness, while cracks do [36]. There are in general only checks on one face of the rotary-cut veneer, the internal side (heart side, loose side), but not on the external face (tight side) [34,37]. There are differences in the amount and depth of the checks, depending on the manufacturing parameters. No checks were found on 0.7 mm-thick peeled veneers, but an increasing number were found on veneers with a greater thickness [38]. Checks facilitate the adhesive to penetrate the wood and enhance mechanical adherence, as mentioned in the previous section. It is known that the later bonding strength can be influenced by the alignment of the veneer lathe checks (checks –checks, checks–no checks, no checks–no checks) [39].

Cooking in boiling water or steaming is often applied before logs are processed to veneer. Veneers are pre-dried before bonding to avoid damages in the board caused by pressurized steam inside the board. Rohumaa et al. [40] used ~3% MC, while an MC of up to roughly 8% is possible for hot bonding, depending mostly on the pressing time, board thickness and the edge–surface ratio.

Further definitions and terms regarding veneer are described in various standards [35,36].

### 3.2. Primary Bonding

In general, numerous veneer sheets are bonded lengthwise to multi-layered boards (LVL) or bonded crosswise (plywood). To improve dimensional stability of LVL, a few veneer sheets are sometimes placed transverse to the main direction. The LVL product “BauBuche Q” has crosswise-oriented veneers to the main direction [41]. At around 150 °C, a two-component phenol-formaldehyde (PF) resin is commonly applied in a hot-pressing process. On both sides of the veneer, the adhesive can be applied with a roller. As a result, a phenolic layer is produced on the board’s surfaces. On the other hand, a one-sided adhesive application is also feasible. Due to the reduced thermal conductivity [20], the temperature gradient is the limiting factor for board thickness. Only a few cm are achievable; for example, 40-mm-thick [42] or 45-mm-thick [43] LVL boards have been manufactured. Longer pressing durations or higher temperatures can be used to achieve high temperatures in the core of the board to cure the adhesive. However, this will result in higher energy consumption and possibly wood degradation. Another approach is to solely utilize cold-cure adhesives for thick beams, as stated in the secondary bonding section. Cold-curing adhesives, on the other hand, are costlier than hot-curing adhesives, and pot time must be taken into account. To make a lengthwise connection, veneers are overlapped a few cm in the longitudinal direction (lap joint). Veneers are sometimes beveled before lap jointing. Surface sanding of single veneer sheets is normally not done on an industrial scale, although it can be done in experiments [40], taking into account the veneer distortion and waviness. A secondary bonding using different adhesives is used to bypass the thickness limitation.

### 3.3. Secondary Bonding

During the secondary bonding process, multi-layered veneer boards are bonded together to thicker plywood or multi-layered LVL beams (LVB). Prior to bonding, cutting the LVL boards into strips (lamellas) is an option. The secondary bonding process is comparable to the glue-laminated and cross-laminated timber bonding processes. The key differences can be seen as a possible compressed surface and a phenol coating on the surface (as described in the primary bonding section), both of which are dependent on the previous hot-bonding process. Commonly applied cold-curing adhesives are (1) emulsion polymer isocyanate (EPI), (2) melamine urea-formaldehyde (MUF), (3) phenol-resorcinol-formaldehyde resin (PRF), and (4) polyurethane (PUR). Depending on the final product application, other adhesives may be utilized. Finger-jointing is a method of joining larger lamellas, and comparable adhesives are frequently applied. Surface manipulation of the LVL lamellas (with or without finger-joints) such as laser treatment, planing, plasma treatment, and sanding [23] is possible and can improve bonding.

## 4. Wood Veneer Acetylation

**Figure 2:**
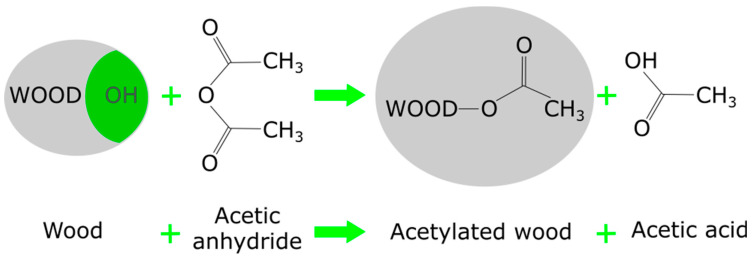
Schematic reaction of a wood hydroxyl group with acetic anhydride to produce acetylated wood and acetic acid as a by-product (own representation based on [44]).

The chemical reaction of veneer acetylation is the same as that of solid wood (Figure 2). Acetic anhydride reacts with reactive wood cell wall hydroxyl groups to form covalent bonds and acetic acid as a by-product [8]. Veneers, on the other hand, are noticeably thinner than solid wood. As a result, the treatment penetration limits are minimized primarily due to (1) its thin thickness, as well as (2) lathe checks, which may promote acetic anhydride penetration into inner portions of veneers.

There has been a lot of research done on veneer acetylation [29,45,46,47,48,49,50,51,52,53,54,55,56,57,58,59,60,61,62,63,64,65,66,67]. Veneers were between 0.1 and 3.5 mm-thick.

Various studies found a wide range of weight percent gain (WPG) for acetylated veneer (Table 1). WPG for rotary-cut veneers and veneers without lathe checks (sawn, sliced) was both high and low, suggesting that lathe checks are only a minor contributor to reach a high WPG.

Alternative acetylation techniques, which are less suitable for solid wood, may also be viable for thin veneers. Acetylation with ketene gas is one such example. According to Rowell [13], the penetration of ketene gas in this procedure was limited to a maximum wood thickness of approximately 3 mm.

## 5. Bonding Acetylated Wood

### 5.1. The Influence of Acetylation on Bonding

#### 5.1.1. Acetic Acid as a By-Product

Acetic acid is a by-product of the acetylation with acetic anhydride. Only small levels of acetic acid remain in the wood once the by-product is removed. However, residual acetic acid affects the wood’s buffering capacity and pH, which can affect the reactivity of certain adhesives [69]. Vick and Rowell [70] assumed that the acetic acid caused an MF resin to gel somewhat after spreading, preventing the bond film from fully forming. The distribution of acetic acid on acetylated veneer is not fully understood yet. Veneers are currently acetylated in stacks rather than as single sheets. As a result, the distribution of residual acetic acid on acetylated veneers within the stacks may vary, potentially affecting bonding. A veneer placed on top of the stack may have a different amount of acetic acid than a veneer placed in the stack’s core. While single veneer acetylation is conceivable on a lab scale, it is not currently used in industrial applications.

#### 5.1.2. Bulking

The acetylated wood stays in a permanently swollen state (bulking) due to the bonded acetyl groups at the cell wall level, which partly occupy the volume accessible to water [8]. Bulked cell walls and, respectively, decreased lumen volumes of acetylated wood were observed by scanning electron microscopy [29]. Bulked wood can still swell to the volume of untreated wood but the swelling is reduced due to the bulking (pre-swollen) [71,72]. The lumen volume depends on the WPG [73].

On untreated veneer, liquid adhesive usually penetrates into open lathe checks. Cell wall bulking has the potential to close open lathe checks. Bulking could also have the opposite effect, increasing the width and depth of the checks. As a result, the width and depth of lathe checks can affect shear strength and wood failure [40]. Additionally, the adhesive penetration can be altered as a result. This could have a detrimental or positive impact on mechanical adhesion. However, the impact of bulking on lathe checks is still not properly understood yet.

#### 5.1.3. Changed Chemistry

According to Bongers et al. [74], the substituted hydroxyl groups (see Figure 2) have an impact on bonding. They further suggest that extractives that have been reallocated throughout the acetylation process may interact chemically with adhesives. In the presence of water or water-soluble adhesives, high-temperature bonding, as described in the primary bonding section, may cleave bonded acetyl groups, leaving acetic acid, acetate, acetyl-, and hydroxy groups in the wood. This putative deacetylation should be further investigated.

#### 5.1.4. Dimensional Stability

The reduced wood–water interaction after acetylation comes along with improved dimensional stability, often expressed as the Anti-Swelling-Efficiency (ASE). This effect was observed by many researchers (for example [18,75,76,77]). Improved dimensional stability also has a great impact on bonding. Moisture-induced swelling and shrinking generate strains on the wood bonding [78]. The adhesive strives to resist the swelling of the wood, resulting in a high concentration of tension around the interface (Figure 1) [22]. These strains are reduced in acetylated wood as compared to references.

Because of the dry and wet cycles, an ASE test with veneer-based products is in some ways also a delamination test. Moreover, veneer products can have a set of thickness recovery (springback) depending on the compression rate. Furthermore, after contact to liquid water, single layers can shift a few cm on the edges, primarily in width but also in length. Therefore, ASE measurements can be influenced by delamination, thickness recovery, and uneven edges, which can lead to inaccuracies. At the very least, this should be considered during an ASE of veneer products and the subsequent interpretation of results.

#### 5.1.5. Moisture Content

As a result of acetylation, the fiber saturation point (FSP) [13] and the equilibrium moisture content (EMC) are reduced. For instance, Čermák et al. [75] found recently highly reduced wood–water interactions of acetylated beech wood as compared to references testing dynamic water vapor sorption, EMC, water soaking, and swelling kinetics. Additionally, Himmel and Mai [71] showed recently that acetylation reduces adsorption and desorption of water and its corresponding EMC, equilibrium times as well as hysteresis in the hygroscopic range of wood. The reduced wood–water sorption in the range of 0–20% RH was especially attributed to the blocking of OH-groups, while the constant reduction in EMC in the range of 20–95% RH, leading to a constant EMC ratio with the untreated control, was only attributed to cell wall bulking [71]. The reduced MC has a high impact on the bonding:

- Large amounts of water are discharged (polycondensation) when hot bonding veneer-based products with PF. Low-MC veneers have a benefit when bonding at high temperatures since they have less vapor pressure and cracks are less likely through the boards. Thereby, the surface-to-edge-ratio of the boards is critical. However, acetylated veneers have a very low MC but can also absorb less water. Consequently, the water movements during hot-bonding acetylated veneer products are not fully understood yet.
- Heat transfer from the top layer to the core during high-temperature pressing is expected to be lower for acetylated veneer products than for references. Bavaneghi and Ghorbani [79] discovered that acetylated particle boards showed reduced heat transfer compared to references, when pressed at 175 °C. Additionally, because of the restricted heat transfer, it may take longer for hot-bonding processes to reach a particular temperature inside a board’s core.
- The penetration of water-soluble adhesive is altered because acetylated wood absorbs less water. Swelling produced by cold curing adhesives absorbing water can cause cracks [25,80], which would be decreased on acetylated wood. On the other hand, the reduced penetration may have a negative impact on mechanical adhesion.
- The compressibility of acetylated wood is affected by the decreasing material moisture [81]. After acetylation, 20 mm-thick (radial) pine (*Pinus radiata* D. Don) samples were densified by only 6.9%, compared to 10% for untreated samples. The reduced compressibility can cause issues, particularly during lap jointing (bumps), as discussed in the primary bonding section.
- Water is required for the chemical reaction of some adhesives, particularly cold-curing 1-C PUR adhesives [82]. Such adhesives absorb water largely from the moisture in the wood, as well as from the humidity in the air. The bonding may fail because of the lower MC. To solve this problem, water can be sprayed on the applied adhesive or on the unwetted side of the workpiece. Longer open waiting times can enable the adhesive to moisten. High humidity may also aid in the resolution of this issue.

#### 5.1.6. Mechanical Performance

Acetylation of wood can increase and decrease the mechanical performance of wood at the same time [18,83]. Increasing factors are higher densities and decreased MC. Two causes of lowered mechanical properties are (1) the reduced amount of fibre and lignocellulose due to the permanent swollen state and (2) degradation of cellulose, hemicellulose and lignin due to the hydrothermal process, stated by the same authors. The hydrothermal effects are more pronounced in the board’s center than on the surface [83]. The degree to which such hydro-thermal effects occur on a single veneer sheet compared to the inner regions of thick solid wood is of great interest. Cracks on thin veneers can be caused by improper handling and transportation, which can degrade the mechanical performance of veneer-based products. Furthermore, acetylation can lead to enhanced hardness [74,81], which can influence finger-jointing and other bonding processes. When compared to untreated samples, acetylated samples perform often better in moist environments. Bongers et al. [74] discovered that acetylated radiata pine (ACCOYA^®^) had higher wet shear strength (8.7–11.7 N/mm^2^) than untreated (3.9–4.3 N/mm^2^). Table 3 contains further information on bonding shear strength and wood failure in wet conditions.

#### 5.1.7. Surface Properties

The surface of wood and its interphases have an effect on the adhesive’s adhesion to the wood [21]. The surface energy and contact angles of acetylated wood are altered [63,84,85], affecting the wood–adhesive interaction.

There are numerous methods for manipulating the surface of wood. These methods are best suited for secondary bonding but can be also useful for primary bonding. The focus of surface manipulation is to improve adhesion by altering the surface. Among other modifications, Lütkemeier [23] compared laser-treated, planed (sharp and blunt), plasma-treated, and sanded surfaces of acetylated wood to untreated references. In comparison to references, the surface topography of acetylated wood remained dissimilar even after equivalent surface manipulation. However, sanding, for example, enhanced the bonding of acetylated wood [86]. The use of primer can also improve the bonding [87].

Surface properties such as pH are covered above in the section on acetic acid as a by-product.

### 5.2. Overview of Bonded Acetylated Material and Products

Even though a variety of acetylated veneer-based products have been produced (Table 2), little is known about their bonding performance (shear strength, wood failure percentage, and delamination); instead, other tests were usually conducted. Acetylation increased dry bonding shear strength very slightly or not at all, while acetylated veneer had a higher wood failure percentage (100%) than untreated controls (33%) [49]. Slabohm and Militz [59] discovered that acetylated beech (*Fagus sylvatica* L.) LVL had somewhat slightly reduced dry shear strength but increased shear strength in wet conditions, notably after high stress in a boiling–drying–boiling cycle. The wood failure was almost unaffected; however, some acetylated samples seemed to perform better under wet conditions. Using a peeling test for pressure-sensitive adhesives, Kiguchi [54] discovered that acetylated samples had lower bonding shear strength than untreated controls.

Further research into the bonding performance of acetylated veneer to veneer products, particularly for load-bearing purposes, is needed.

The bonding performance of acetylated solid wood was studied in greater depth (Table 3). Because different wood species, adhesives, and acetylation procedures (industrial and lab-scale) were utilized to bond acetylated wood, comparing results from different studies is complex. Acetylated wood appeared to perform better in wet conditions and was occasionally somewhat slightly higher in dry settings. The adhesive product and its proper application might be considered as the key driver of the bonding. For example, even though identical conditions of acetylated wood were utilized, EPI, PRF, PVA, and RF adhesives showed already equal, greater, or lower shear strength [88].

Finger-jointed acetylated wood was also investigated. Papadopoulos [92] used polyvinyl acetate (PVA) to bond acetylated beech (*Fagus sylvatica*). Compared to untreated controls, the MOE and MOR were both lower. They concluded that the acetylation procedure has a negative impact on the mechanical performance of wood; therefore, MOE and MOR of the acetylated wood were lowered. As previously stated, acetylation has the ability to both improve and degrade mechanical properties. In addition, the study contains no information of MOE and MOR of acetylated solid wood. Therefore, the poor mechanical performance of acetylated finger-jointed wood could also be caused by a poor bonding performance and not solely due to lowered mechanical performance of the acetylated wood.

The majority of the bonding findings originate from acetylated solid wood. Bonding veneers, on the other hand, is different. Solid wood usually just requires one cold-bonding process, although veneer-based products typically require up to two bonding processes, as previously mentioned. Furthermore, veneers feature lathe checks that aid adhesive penetration, resulting in a distinct mechanical adhesion. Additionally, rotary cut veneers have a unique plane of cut. Therefore, there is further research required on acetylated veneer-based products.

## 6. Acetylated Wood for Structural Purposes and Acetylated Veneer Products on the Market

The load-bearing capability of acetylated solid wood has already been demonstrated. In 2008 and 2010, two heavy-load-bearing traffic bridges were erected in Sneek, the Netherlands [93,94]. Several pedestrian bridges and column-like structures (use class 3) have been built in the last decade [69]. Blaß et al. [95] showed how to produce glued laminated timber suited for exposed structures. Because load-bearing construction in exterior use is one of the toughest conditions for wood in application, a strong bonding was required to create these wood products. This demonstrates that strong bonds can be formed with acetylated wood.

Even though durability and dimensional stability are improved after acetylation, no relevant acetylated veneer products are available on the market up to date. However, many veneer-based products are well-established on the market, for example BauBuche, dehonit^®^, Compreg™, Impreg™, Delignit^®^, Feinholz^®^, Festholz^®^, Kerto^®^, Panzerholz^®^, VANyCARE^®^ and Pagholz^®^.

Generally, like with all new modification technologies, there are challenges to overcome by launching new wood modification products on the market. Militz and Lande [96] stated that amongst the most important challenges are non-existing upscaled processes and new products with unknown properties for the users. Another explanation for the lack of market introduction is that it is not a matter of material performance, but rather of the wood industry’s established and rigid structure [97]. Further, often new specialized wood modification companies, as well as the formation of new networks within existing structures, are required for a successful implementation of chemical wood modification technologies, as stated by the same author. Another technical problem is that acetylation of veneer, and the production of veneer-based products are two separate processes. Other wood modification technologies, such as impregnation modification with resins, typically combine curing and hot bonding.

However, acetylation modification is already successfully implemented on the market (ACCOYA^®^ and TRICOYA^®^), as are veneer-based productions with other modifications (Impreg™ as an example). Therefore, an implementation of a relatively simple modification technology, such as veneer acetylation, can be expected in the near future, merging existing acetylation and veneer production techniques.

## 7. Conclusions and Outlook

Acetylation is a well-known method for improving wood’s dimensional stability and durability. Although there has been a lot of research regarding veneer acetylation, the acetylated veneers were rarely glued to veneer-based products and bonding performance was tested on rare occasions. The bonding performance of acetylated solid wood was found to be quite variable, depending on the adhesive type and application, as well as the wood species and acetylation method. Although it was difficult to evaluate, it appears that acetylated veneer products perform better in wet conditions, which is also beneficial for the bonding.

With regard to the bonding of acetylated veneer-based products, we discovered multiple research needs, mainly:

- How bulking affects lathe checks, cracks, and adhesive penetration;
- If high temperatures during bonding alter the stability of bonded acetyl groups (deacetylation) under the presence of water-soluble adhesives;
- If densification is still possible or only hampered;
- Performance of primary and secondary bonding, besides finger- and lap-jointing.

Currently, much research into the acetylation of veneer is ongoing. In our own cooperative projects are manufacturing processes of LVL-developed acetylated beech (*Fagus sylvatica*). Our main project on veneer acetylation is in cooperation with the University of Kaiserslautern and several industry partners such as Accsys Technologies, the Netherlands; Deutsche Holzveredelung Schmeing GmbH & Co. KG, Germany; and Schaffitzel Holzindustrie GmbH & Co. KG, Germany. Aside from our projects, research into the usage of acetylated veneer for battery housings and other automobile parts is ongoing.

In the future, an implementation of a relatively simple modification technology, such as veneer acetylation, can be expected. To show the potential of acetylated beech LVL, a wooden lookout tower (**alp**turm, 61.5 m) is planned to be built soon in Heubach, Germany.

## Figures and Tables

**Table 1:** The WPG ranked according to maximal values. Additional information is included, such as wood species, veneer thickness, and veneer manufacturing process (n.a. = no information available or not discovered). Yang et al. [65] compared thermal and microwave liquid phase and vapor phase reactions.

WPG [%]		Wood Species	Thickness [mm]	Manufacturing Process	Reference
Max.	Min.				
14.5	8.2	Maple (*Acer mucrophyllum* Pursh)	0.8	sliced	[46]
16	6	Radiata pine (*P. radiata* D. Don)	2	rotary cut	[45]
17	15	Douglas fir (*Pseudotsuga menziesii*)	3	n.a.	[56]
18.1	4.1	Scots pine (*Pinus sylvestris* L.) sapwood	0.1	micro veneer	[50]
18.3	1.5	Spruce (*Picea jezoencis* Carr.)	3.5	rotary cut	[51]
20.0	13.5	Oriental beech (*Fagus orientalis* Lipsky)	2	rotary cut	[55]
20	7	Spruce (*Picea jezoencis* Carr.) Larch (*Larix leptolepsis* Gord.) Douglas fir (*Pseudotsuga menziesii* Franco)	3	rotary cut	[52]
20.5	16.6	Sugi (*Cryptomeria japonica* D. Don: Japanese cedar)	0.2	heart-sliced (quartersawn)	[54]
22	3	Scots pine (*Pinus sylvestris*) European lime (*Tilia vulgaris*)	0.1	micro veneer	[57]
22	20	Birch (*Betula pendula*; *Betula pubescens*)	1.5	rotary cut	[64]
22.9	20.9	Radiata pine sapwood (n.a.)	1.3	rotary cut	[47]
23.2	5.8	Scandinavian Scots Pine (*Pinus sylvestris* L.) sapwood	0.1	micro veneer	[68]
23.4	12.4	Sugi (*Cryptomeria japonica* D. Don)	3	sliced	[65]
24.1	6.5	Spruce (*Picea abies* (L.) Karst)	2	n.a.	[58]
24.5	6.9	Sugi (*Cryptomeria japonica* D. Don)	3	sliced	[65]
24.9	23.9	Beech (*Fagus sylvatica* L.)	2.3	rotary cut	[59]
25.1	9.1	Sugi (*Cryptomeria japonica* D. Don)	3	sliced	[65]
26.7	0.4	Spruce (*Picea abies* (L.) Karst)	1, 2	n.a.	[61]

**Table 2:** Bonding of acetylated veneer—An overview of selected acetylated veneer-based products (phenol-formaldehyde (PF); phenol-resorcinol-formaldehyde (PRF); resorcinol-formaldehyde (RF); urea-formaldehyde (UF)).

Veneer Based Product	Adhesive	Reference
8-layered LVL	PF and PRF	[59]
6-layered LVL	RF	[52]
plywood	PRF (acetylated) and PF (reference)	[64]
plywood	UF resin	[55]
2-layered specimen	soy-protein based resin	[49]
2-layered specimen	PF	[46]
top layers on solid wood	PRF	[47]
top layers on low-density particleboards	isocyanate resin	[56]
top layers on wood plastic composites (WPC)	adhesive free approach	[45]
pin-block for acoustic tests	RF	[66]

**Table 3:** Compared to references, the bonding performance (shear strength, wood failure percentage, and delamination) of acetylated wood was: (↑) better; (=) equal; (↓) worse; (x) not tested; additionally, combinations are conceivable, such as when several adhesives were applied. Emulsion polymer isocyanate (EPI), melamine-urea-formaldehyde (MUF), melamine-formaldehyde (MF), phenol-formaldehyde (PF), phenol-resorcinol-formaldehyde (PRF), polyurethane (PUR), polyvinyl acetate (PVA), resorcinol-formaldehyde (RF), and urea-formaldehyde (UF).

Wood Species	Method	Adhesive	Shear Strength		Wood Failure		Delamination	Reference
			Dry	Wet	Dry	Wet		
Rubberwood (*Hevea brasiliensis* Müll. Arg.)	EN 302-1:2013	PUR	=	↑	=	↑	×	[89]
		MUF	↑	↑	↓	↓	×	
		PRF	=	↑	↑	=	×	
Radiata pine (*Pinus radiata*)	EN-302-2:2013	MUF	×	×	×	×	↓	[87] ^1^
		MUF + RF primer	×	×	×	×	↓	
Beech (*Fagus sylvatica*)	EN-302-2:2013	MUF + RF primer	×	×	×	×	↓	
Yellow-poplar (*Liriodendron tulipifera*)	ASTM Method D905, ASTM D5266-99	RF	=	↑	=	=	×	[90] ^2^
		EPI	=	↓	↓	↓	×	
		epoxy	=	↑	=	↑	×	
Radiata pine and Scots pine (*Pinus sylvestris*)	IFT Richtlinie HO-10/1, BRL 2902, CEN/TS 13307-2	PUR 1	=	↑	=	↑	=	[74] ^3^
		PUR 2	=	↑	=	=	=	
		PUR 3	=	↑	=	↑	=	
Yellow-poplar sapwood (*Liriodendron tulipifera*)	ASTM D 905-03, ASTM D 5266-99	RF	↑	↑	↑	=	×	[91]
		MF	=	↑	↓	↓	×	
		epoxy	↑	↑	=	↑	×	
		EPI	=	↓	↓	↓	×	
Yellow poplar sapwood	ASTM D 905-86	EPI (A)	=	=	=	↓	×	[70] ^4^
		EPI (C)	↑	↓	=	↓	×	
		PUR	=	↓	=	↓	×	
		PUR hot-melt	=	=	=	=	×	
		PVA	↓	↓	=	=	×	
		PVA cross-link	=	=	=	↓	×	
		neoprene contact-bond	=	=	=	=	×	
		waterborne contact-bond	=	=	=	=	×	
		casein	↓	↓	=	=	×	
		epoxy	=	↓	=	=	×	
		MF	↓	↓	=	↓	×	
		urea-formaldehyde hot set	=	↓	=	↓	×	
		urea-formaldehyde cold set	↓	↓	↓	↓	×	
		RF cold set	=	↓	↑	=	×	
		PRF cold set	=	=	↑	↓	×	
		PRF hot set	=	↓	=	↓	×	
		PF	=	↓	=	↓	×	
		PF acid-catalysed	=	=	=	=	×	

^1^ PRF has been used to bond acetylated radiata pine samples (0.22% delamination) and acetylated beech samples (0% delamination); however, no references were bonded. ^2^ We solely compared unplaned acetylated samples in comparison to unplaned untreated samples. The research also includes data on acetylated planed wood. ^3^ Six PUR adhesives, one MUF adhesive, and one PVA adhesive were used in total. These adhesives were also put through tests in accordance with IFT Richtlinie HO-10/1, BRL 2902, and CEN/TS 13307-2. The presented results just indicated whether or not the test had been passed. As a result, only three PUR adhesives were evaluated in this table, with further information provided. ^4^ Comparing untreated references (0% WPG) to the highest WPG (20%).
